# circPTN Promotes the Progression of Non-Small Cell Lung Cancer through Upregulation of E2F2 by Sponging miR-432-5p

**DOI:** 10.1155/2022/6303996

**Published:** 2022-09-20

**Authors:** Jiayuan Su, Jinrong Zhou, Yachan Feng, Haojie Zhang, Xinyu Zhang, Xiaorong Zhao, Yong Li, Xueling Guo

**Affiliations:** ^1^Department of Cardiothoracic Surgery, Xichang People's Hospital, No. 169 Shunhe Road, Xichang, 615000 Liangshan Yi Autonomous Prefecture, Sichuan Province, China; ^2^Department of respiratory and critical care medicine, People's Hospital of Dongxihu District, Wuhan, Hubei 430040, No. 48, No. 1, Jin Bei Road, Dongxihu District, Wuhan, Hubei, China; ^3^College of Food Science & Biology, Hebei University of Science and Technology, Shijiazhuang, China

## Abstract

**Background:**

Non-small cell lung cancer (NSCLC) is one of the most prevalent cancers, accounting for around 80% of total lung cancer cases worldwide. Exploring the function and mechanism of circRNAs could provide insights into the diagnosis and treatment for NSCLC.

**Methods:**

In this study, we collected tumor tissues and adjacent normal tissues from NSCLC patients to detect the expression level of circPTN and analyzed the association of its expression level with the clinicopathological parameter of NSCLC patients. Moreover, the functional engagement of circPTN in NSCLC cells was examined by cell counting kit-8 (CCK-8) cell proliferation assay, transwell migration and invasion assays, and tube formation assay. Quantitative real-time polymerase chain reaction (qRT-PCR) and Western blotting (WB) analysis were used to detect gene and protein expression, respectively. The molecular targets of cicrPTN were predicted using starBase online resources, which was validated by RNA immunoprecipitation (RIP) and dual-luciferase reporter assay.

**Results:**

Compared with adjacent normal tissues, there was a remarkable increase of the circPTN levels in NSCLC tissues. A high level of circPTN expression was associated with more lymph node metastasis (LNM) and advanced TNM stages. Functionally, circPTN knockdown inhibited the proliferation, migration, and invasion and tube formation ability of NSCLC cells. We further demonstrated that circPTN regulated the malignant phenotype of NSCLC cells through targeting the miR-432-5p/E2F2 axis.

**Conclusion:**

Together, our results suggest that circPTN, which is upregulated in NSCLC tissues, could serve as a prognostic marker for NSCLC patients. circPTN regulates the malignant progression of NSCLC cells through targeting the miR-432-5p/E2F2 axis, which may be employed as a potential strategy for the management of NSCLC.

## 1. Introduction

Lung cancer has become one of the most common cancers globally [1], with an increasing incidence in recent years [2]. In the meantime, lung cancer has become one of the main cancer-related mortalities globally. Non-small cell lung cancer (NSCLC) is the main subtype of lung cancer, accounting for around 80% of total lung cancer cases [3]. Despite the development of various treatment modalities, the overall survival (OS) is unsatisfactory, especially in patients with metastasis, with a 5-year OS rate less than 5% [4]. Exploring the markers for early diagnosis and prognosis prediction and investigating the mechanisms in its progression are of great important for the effective management of NSCLC in clinics.

Unlike linear RNAs, circRNAs have a closed-loop structure, rendering them high stability and resistance to the degradation by exonucleases [5]. circRNAs frequently serve as molecular sponges for other noncoding RNAs such as micro-ribonucleic acid (miRNA), which in turn regulates the activity of miRNAs and the expression of the downstream target mRNAs [6]. circRNAs have been implicated in regulating the initiation or progression of many pathological conditions [7], such as cancers [8], atherosclerosis (AS), neurological diseases, endocrine and metabolic diseases, and viral infections [9]. Due to its stable circular structure [10], circRNAs are highly abundant in eukaryotic cells, and their expression pattern shows tissue-specificity [11]. Recently, 257 novel circRNAs were identified in the samples of patients with colorectal cancer [12], indicating that cancer-specific expression of cicrRNAs play critical roles in cancer progression. Another study demonstrated that serum circ-KLDHC10 level in HCC patients were significantly elevated compared with healthy subjects [13], which may serve as a diagnostic marker. In contrast, hsa_circ_0000190 showed significant downregulation in plasma samples in patients with gastric cancers [14]. Interestingly, a recent study indicates that there was a significant difference in the expression of circRNAs in patients with gastric cancer (GC) before and after surgery [15]. In addition, circRNAs are also implicated in the initiation and progression of multiple types of cancer [16–18]. Moreover, different circRNAs deregulated in NSCLC have been proposed as diagnostic markers for the management of NSCLC patients [19–21], indicating a clinical value of circRNA investigation.

miRNAs are a class of short (average 22 nucleotides) endogenous non-coding RNAs with critical roles in post-transcriptional gene regulation in cancer development and progression [22, 23]. miRNAs are able to regulate the expression of oncogenes or tumor suppressor genes by competitively biding to the seed sequences in the untranslated region (UTR) of target mRNAs, ultimately inducing target mRNA degradation or translational arrest [24]. A previous report indicated that miR-432-5p was downregulated in lung cancer, suggesting its tumor suppressor function [25]. It was reported that circPTN functions as an oncogene in glioma and liver cancer [25, 26] by targeting miR-432-5p. However, the functional roles of circPTN and miR-432-5p in NSCLC remain unclear.

Considering these aspects, in this study, we attempted to unveil the expression pattern and functional role of circPTN and miR-432-5p in NSCLC. We demonstrated the upregulation of circPTN in NSCLC tissues and studied its functions in NSCLC cells by cell proliferation assay, transwell migration and invasion assays, and vessel formation assay. Furthermore, the mechanistic interactions among of circPTN, miR-432-5p, and E2F2 (E2F Transcription Factor 2) were predicted by starBase and investigated by RNA immunoprecipitation (RIP) and dual-luciferase reporter assays. Overall, our data shed light on the role of circPTN/miR-432-5p/E2F2 axis in regulating the malignancy of NSCLC cells, which provides insights into the diagnosis, treatment, and prognosis assessment of NSCLC.

## 2. Materials and Methods

### 2.1. Tumor Samples and Ethical Approval

A total of 90 NSCLC samples and paired para-tumor tissues in NSCLC patients were collected by surgery from October 2019 to January 20121 in the Xichang People's Hospital. All the enrolled patients had not gone through chemotherapy or radiotherapy. Prior to sample collection, informed consent was provided by each patient. All the experimental procedures were approval by the Ethics Committee of Xichang People's Hospital.

### 2.2. Cell Culture

Human bronchial epithelial (HBE) cells and NSCLC cells (A549, CALU3, CALU6, H1229, and H1650) were obtained from Beyotime (Hangzhou, China) and Cell Bank of the Chinese Academy of Sciences (Shanghai, China). All the cells were cultured in the RPMI-1640 medium (Gibco, Rockville, MD, USA) supplemented with 10% fetal bovine serum (FBS, Gibco, Rockville, MD, USA) and 100 U/ml of penicillin and 100 *μ*g/ml of streptomycin in a humidified incubator containing 5% CO_2_ at 37°C.

### 2.3. Cell Transfection

Cells were inoculated within the 6-well plates until 75% confluence. Cell transfection was performed using Lipofectamine™ 2000 reagent (Invitrogen, Carlsbad, CA) or Entranser™-R4000 (Engreen Biosystem, Beijing, China) according the manufacturer's instructions. 10 *μ*L of Lipofectamine 2000 and 10 *μ*L of siRNA-SNHG12 or siRNA-NC (RiboBio Co. Ltd., Guangzhou, China) were mixed in 500 *μ*L of serum-free medium for 15 min. For plasmid, 4 *μ*l of Entranser™-R4000 reagent and 2.68 *μ*g of pcDNA-SNHG12 or pcDNA empty vector (GenePharma, Suzhou, China) were mixed in 500 *μ*L serum-free medium for 15 min. After the incubation, the mixture was added into each well dropwise and the cells were incubated for 6 h. The medium was replaced with fresh culture medium after 6 h of transfection, and the cells were subjected to further experiment after 48 h.

### 2.4. Quantitative Real-Time Polymerase Chain Reaction (qRT-PCR) Analysis

Total RNA samples were extracted using miRNeasy mini kit (QIAGEN, Hilden, Germany). An miRNA RT kit (Applied Biosystems, Foster City, CA, USA) was utilized for reverse transcription. qRT-PCR was performed using SYBR Green Master Mixture (Roche, Basel, Switzerland) on the 7500 Fast Real-Time PCR System (Applied Biosystems, USA). 2^-*ΔΔ*Ct^ method was adopted for relative gene expression analysis, with GAPDH and U6 being the endogenous references. The primer sequences (Genewiz, South Plainfield, NJ, USA) are as follows (from 5'–3'): circPTN-forward: TCAAGAATGCAGGCTCAAC, reverse: TCAAGAATGCAGGCTCAAC; miR-432-5p-forward: AACGAGACGACGACAGACT, reverse: CTTGGAGTAGGTCATTGGGT; E2F2-forward: CGTCCCTGAGTTCCCAACC, reverse: GCGAAGTGTCATACCGAGTCTT; GAPDH-forward: CTGGGCTACACTGAGCACC, reverse: AAGTGGTCGTTGAGGGCAATG; and U6-forward: CTCGCT TCGGCAGCACA, reverse: AACGCT TCACGA ATTTGCGT. Thermal cycling conditions for qPCR were as follows: 95°C for 2 minutes, 40 cycles of 95°C for 30 seconds, 60°C for 30 seconds, and 72°C for 60 seconds.

### 2.5. CCK-8 Proliferation Assay

Cell proliferation was examined using the CCK-8 assay (Beyotime, C0037). Initially, the cells (2 × 10^3^ cells/well) were seeded into the 96-well plate and incubated for 0, 24, 48, and 72 h. At indicated time points, 100 *μ*L of 10% CCK-8 working solution was added to the wells for 2 h of incubation at 37°C in 5% CO_2_. The absorbance values of different groups were measured at 450 nm using the microplate reader (BioTeke, Winooski, VT, USA).

### 2.6. EdU Incorporation Assay

The proliferation of cells was determined using the EdU staining proliferation kit (KeyFluor488 EdU Kit, keyGEN BioTECH, Jiangsu, China), following the manufacturer's instructions. Cells were seeded in 96-well plates (1 × 10^4^ cells/well) in 100 *μ*L medium. The next day, the cells were cultured with 20 *μ*M EdU for 2 h under 5% CO_2_ and 37°C. Then, 4% paraformaldehyde was added to fix the cells for 30 min, and 0.5% Triton-X-100 in PBS was added and incubated for 20 min. After the removal of the solution, 1x Click-iT® reaction cocktail was added to cells and incubated for 30 min. The staining cocktail was removed and cells were counter-stained by 500 nM DAPI in PBS and the images were captured under a Leica AM6000 microscope (Leica, Wetzlar, Germany).

### 2.7. Transwell Migration and Invasion Assay

Cell invasion and migration were assessed by transwell assay. Briefly, cells with different treatments were trypsinized and resuspended in serum-free medium. The transwell upper chamber (Corning, New York, USA) without Matrigel (BD Biosciences, MA, USA) was used for migration assay, while transwell chamber coated with Matrigel was used for invasion assay. A total of 2.5 × 10^5^ cells were seeded into the upper chamber in 400 *μ*L serum-free medium, and 500 *μ*L of 20% serum-containing medium was added to the lower chamber. After 24 hours, cells on the membrane were fixed with 3.7% paraformaldehyde for 10 min and stained with 0.5% crystal violet (Sigma, Germany) for 20 min. Cells were photographed under a Leica AM6000 microscope.

### 2.8. Tube Formation Assay

Tube formation was performed using an in vitro angiogenesis assay kit (ab204726; Abcam). Initially, cells (2.0 × 10^4^) were seeded in a 96-well plate coated with 50 *μ*l of extracellular matrix (ECM) solution. After 18 h, cell morphology was observed with a phase-contrast microscope (DMI6000B; Leica, Wetzlar, Germany). The Image J Angiogenesis Analyzer (National Institutes of Health (NIH), Bethesda, MD, USA) was used for quantification of the network structure.

### 2.9. RNA Pull-Down Assay

Cell lysates were collected using IP lysis buffer (Beyotime, P0013), with 10% total lysate saved as input. 100 nM of biotin-labeled control probe or wild-type (WT) circPTN probe was mixed with cell lysate and incubated for a 12 h at 4°C. 100 *μ*L of Dynabeads M-280 Streptavidin (Thermo Fisher Scientific, CA, USA) was added to the solution and incubated for 2 h under 4°C. The beads were precipitated using a magnetic bar and washed three times using high salt buffer (500 mM NaCl, 1% Triton X-100, 0.1% SDS, 20 mM Tris-HCl, pH 8.0, 2 mM EDTA). Finally, the precipitated RNA samples or RNA in the input samples were purified using the TRIzol reagent (Invitrogen, Carlsbad, CA, USA) for qRT-PCR assay.

### 2.10. Dual-Luciferase Reporter Assay

The sequence containing the wild-type binding site or the sequence with mutated binding site was cloned into the PmirGLO vector expressing firefly luciferase (Promega, WI, USA). The reporter plasmid and Renilla luciferase (hRlucneo) control plasmid were co-transfected into cells using Lipofectamine^TM^ 2000 (Invitrogen). 48 h after the transfection, the relative luciferase activities were measured using dual-luciferase reporter assay kit (Promega, WI, USA) on a luminescence GloMax® discover microplate reader (Promega). Firefly luciferase activity in each sample was normalized to that of Renilla luciferase.

### 2.11. Western Blot Assay

RIPA buffer (Beyotime Biotechnology, Shanghai, China) was utilized to extract total protein, and protein concentration was determined using a BCA protein assay kit (Beyotime Biotechnology, Shanghai, China) as per instructions. The protein samples were separated by sodium dodecyl sulfate-polyacrylamide gel electrophoresis (SDS-PAGE) and transferred onto PVDF membranes (Millipore, Billerica, MA). After blocking with the skimmed milk, the membrane was incubated using the following antibodies: E2F2 (Ab138515, Abcam), actin (ab8227, Abcam), E-cadherin (ab231303, Abcam), N-cadherin (ab76057, Abcam), vimentin (ab137321, Abcam) at 1 : 1000 dilutions at 4°C overnight. The membrane was washed with the TBST buffer and then incubated with HRP-conjugated secondary antibody under ambient temperature for 2 h. The protein bands were visualized using the ECL detection kit (Yeasen, Shanghai, China), with actin as the internal control.

### 2.12. Xenograft Tumor Models

All the animal experiments were approved by the Animal Use and Care Committee of Hebei University of Science and Technology and carried out according to the institutional regulations and guidelines. Briefly, the female BALB/c nude mice (4-week-old) were randomly assigned into 2 groups (*n* = 6 in a group): 1 × 10^6^ cells stably transfected with sh-NC or cell stably transfected with sh-circPTN by subcutaneous injection on the flank of the mice. Tumor volume was determined at 7-day intervals, with the following formula: *V* (tumor) = 0.5 × length × width^2^. On day 28, the mice were euthanized by carbon dioxide asphyxiation and cervical dislocation. The tumor samples were removed for histological and IHC analysis.

### 2.13. Immunohistochemistry (IHC)

Tumor tissues were fixed with 4% paraformaldehyde (PFA, V/V), followed by paraffin embedding. 4-*μ*m tissue sections in paraffin were soaked in xylene for 15 min before dehydration with gradient ethanol. Each section was then soaked with citric acid (pH 6.0 DAKO) for 10 min at 95° for antigen retrieval and cooled to ambient temperature. After washing with TBST buffer for a 15-min period, the sections were incubated with 3% H_2_O_2_ for 10 min. Tissue sections were blocked using 5% bovine serum albumin (BSA) for 30 min, followed by overnight incubation using primary antibodies E2F2 (Ab138515, Abcam) and Ki67 (ab15580, Abcam) under 4°C. Color development was performed using a DAB color-rendering kit (Soleibol, Japan). The images were captured using a Leica AM6000 microscope.

### 2.14. Histopathological Analysis

H&E staining was performed using the H&E stain kit (ab245880, Abcam). Tissue sections were incubated in hematoxylin solution, Mayer's (Lillie's Modification), for 5 min. The section was rinsed twice with distilled water and incubated with adequate bluing reagent for 30 seconds. After washing with distilled water, the section was dehydrated in absolute alcohol, followed by staining with eosin Y solution for 2 min. The section was rinsed using absolute ethanol for three times and then mounted to a slide, and the images were collected under an inverse microscope.

### 2.15. Statistical Analysis

The data were expressed as means ± SD. Kaplan–Meier (K-M) curve was used to analyze the overall survival in NSCLC patients. GraphPad Prism 5 (GraphPad Software, La Jolla, CA, USA) was utilized for statistical analysis. Student's *t*-test was adopted to compare the difference between two groups, while one-way ANOVA was used to compare the difference among multiple groups. *P* < 0.05 was deemed to be statistical significant.

## 3. Results

### 3.1. circPTN Is Highly Expressed in NSCLC Cells and Tissues

**Figure 1:**
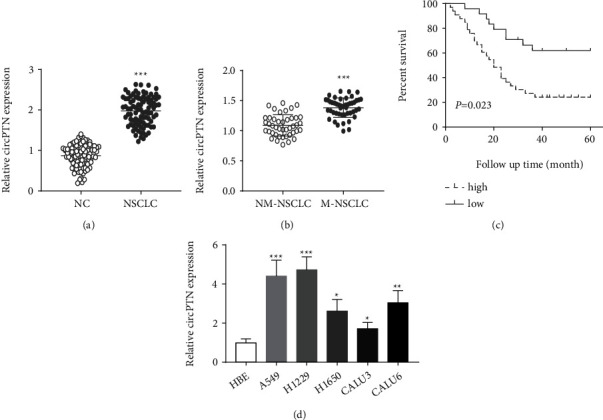
circPTN is highly expressed in NSCLC cells and tissues. (a) circPTN levels were analyzed in NSCLC tissue samples and para-cancerous tissues. (b) circPTN levels were analyzed in non-metastatic tumor samples and metastatic samples. (c) KM plotter analysis of circPTN expression level and the overall survival in NSCLC patients. (d) qRT-PCR was employed to detect the expression levels of circPTN in different NSCLC cell lines. ∗*P* < 0.05; ∗∗*P* < 0.01; and ∗∗∗*P* < 0.001.

Initially, the expression levels of circPTN were compared between NSCLC tissues and para-tumor tissues. qRT-PCR results showed circPTN level was upregulated in NSCLC tissues as compared to the para-cancerous tissues (Figure 1a). Besides, the cicrPTN level was also higher in the patients with metastasis when compared to the ones without metastasis (Figure 1(b)). The patients were divided into circPTN high-expression and low-expression groups based on the median expression value of circPTN. A high circPTN expression was associated with more lymph node metastasis (LNM) and advanced TNM stage (Table 1). Further, as revealed by the KM plotter, high circPTN expression was correlated with a worse overall survival in NSCLC patients (Figure 1(c)). In addition, circPTN expression levels were also significantly higher in NSCLC cells compared to Human bronchial epithelial (HBE) cells (Figure 1(c)). These data indicates that high level expression of circPTN predicts a poor prognosis in NSCLC patients.

### 3.2. Knockdown of circPTN Inhibits Cell Proliferation, Invasion and Migration, and Tube Formation in NSCLC Cells

**Figure 2:**
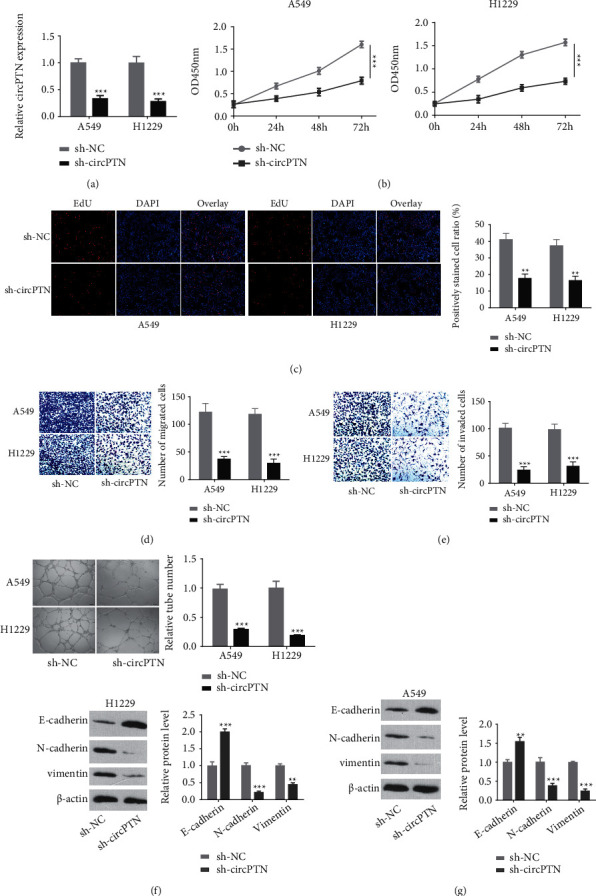
Knockdown of circPTN inhibits the proliferation, migration, invasion, and tube formation ability of NSCLC cells. (a) Knockdown efficiency of sh-circTPN was detected by qRT-PCR. (b) CCK-8 proliferation assay in A549 and H1229 cells transfected with sh-NC or sh-circTPN (c) EdU incorporation assay in A549 and H1229 cells transfected with sh-NC or sh-circTPN. (d) Transwell-migration assay in A549 and H1229 cells transfected with sh-NC or sh-circTPN. (e) Transwell-invasion assay in A549 and H1229 cells transfected with sh-NC or sh-circTPN. (f) Tube formation assay in in A549 and H1229 cells transfected with sh-NC or sh-circTPN. (g) WB was performed to detect EMT-related proteins (E-cadherin, N-cadherin, and vimentin) in A549 and H1229 cells transfected with sh-NC or sh-circTPN. ∗ *P* < 0.05; ∗∗*P* < 0.01; and ∗∗∗*P* < 0.001.

Two NSCLC cell lines (A549, H1229) with the highest circPTN expression (Figure 1(d)) were selected for subsequent experiments. After the transfection of siRNAs, circPTN levels were significantly reduced by sh-circPTN in H1229 and A549 cells (Figure 2(a)). CCK-8 proliferation assay and EdU incorporation assay showed that circPTN knockout significantly inhibited the cell proliferation in both cell lines (Figure 2(b) and 2(c)). As revealed in the Transwell assays, circPTN knockout impaired the migration (Figure 2(d)) and invasion abilities (Figure 2(e)). In addition, tube-forming ability was also undermined upon circPTN silencing (Figure 2(f)). We also examined epithelial–mesenchymal transition (EMT)-related proteins by Western blot. CircPTN silencing increased the expression of epithelial marker E-cadherin, while mesenchymal markers including vimentin and N-cadherin were decreased (Figure 2(g)). Together, these data suggest that circPTN expression is indispensable of the malignant phenotype of NSCLC cells.

### 3.3. circPTN Targets miR-432-5p

**Figure 3:**
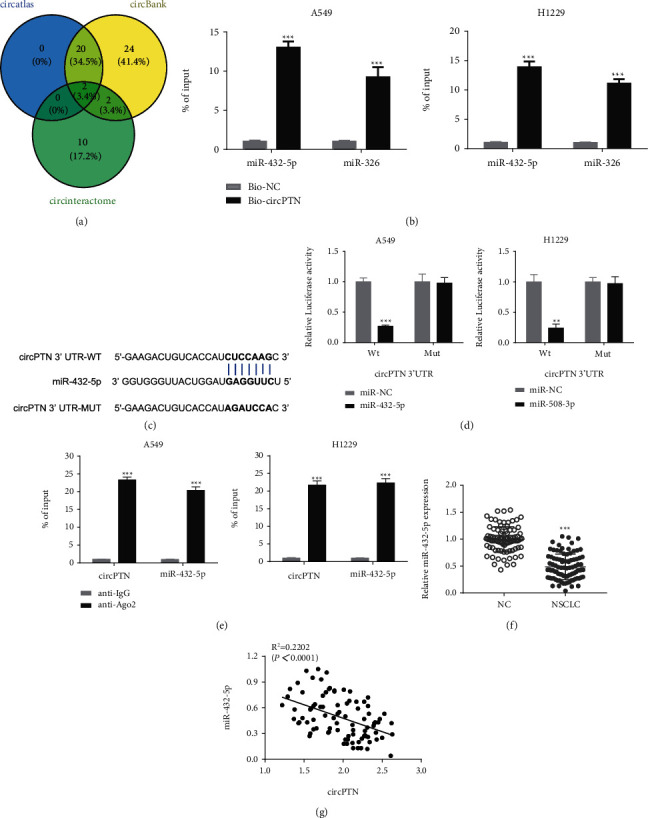
circPTN targets miR-432-5p. (a) Three prediction software tools were used to predict the miRNA targets of circPTN. (b) RNA-pull down assay using biotin-labeled control (Bio-NC) or circTPN probe (Bio-circTPN) in A549 and H1229 cells. The relative enrichment of miR-432-5p and miR-326 was quantified by qRT-PCR. (c) The predicted binding sites between circPTN and miR-432-5p. (d) Dual-luciferase reporter assay in A549 and H1229 cells using WT reporter (with wildtype binding sites) or MUT reporter (with mutated binding sites) in the presence of miR-NC or miR-432-5p mimic. (e) RIP-qRT-PCR assay was performed in A549 and H1229 cells using anti-Ago2 or IgG. (f) qRT-PCR was used to detect the expression of miR-432-5p in 90 pairs of NSCLC tissues and adjacent normal tissues. (g) The correlation between circPTN and miR-432-5p expression levels in NSCLC tissues. ∗*P* < 0.05; ∗∗*P* < 0.01; and ∗∗∗*P* < 0.001.

To search for the downstream miRNA targets, we employed three different software tools (“circAtlas,” “circBank,” and “circInteractome”) to predict the interacting partners of circPTN, which revealed that hsa-miR-326 and hsa-miR-432-5p could be potential targets (Figure 3(a)). RNA pull-down analysis using biotin-labeled circPTN probe showed that both miRNAs were enriched with the circPTN probe, with a much higher enrichment for miR-432-5p (Figure 3(b)). We cloned the potential binding sites (WT) or the mutated binding sites (MUT) between circPTN and miR-432-5p and performed dual-luciferase reporter assay in the presence of miR-432-5p mimic or miR-NC (Figure 3(c)). The results showed that miR-432-5p mimic significantly suppressed luciferase activity of WT reporter, while the mutation of the biding sites abrogated this effect (Figure 3(d)). In addition, RIP-assay demonstrated that Ago2 antibody enriched more circPTN and miR-432-5p when compared with the IgG group (Figure 3(e)). Moreover, miR-432-5p expression level showed a downregulation in NSCLC tissues (Figure 3(f)), and there was a negative correlation between miR-432-5p and circPTN in NSCLC tissues (Figure 3(g)). Together, these data suggest that miR-432-5p is a target negatively regulated by circPTN.

### 3.4. circPTN Regulates E2F2 Protein Expression through Sponging miR-432-5p

**Figure 4:**
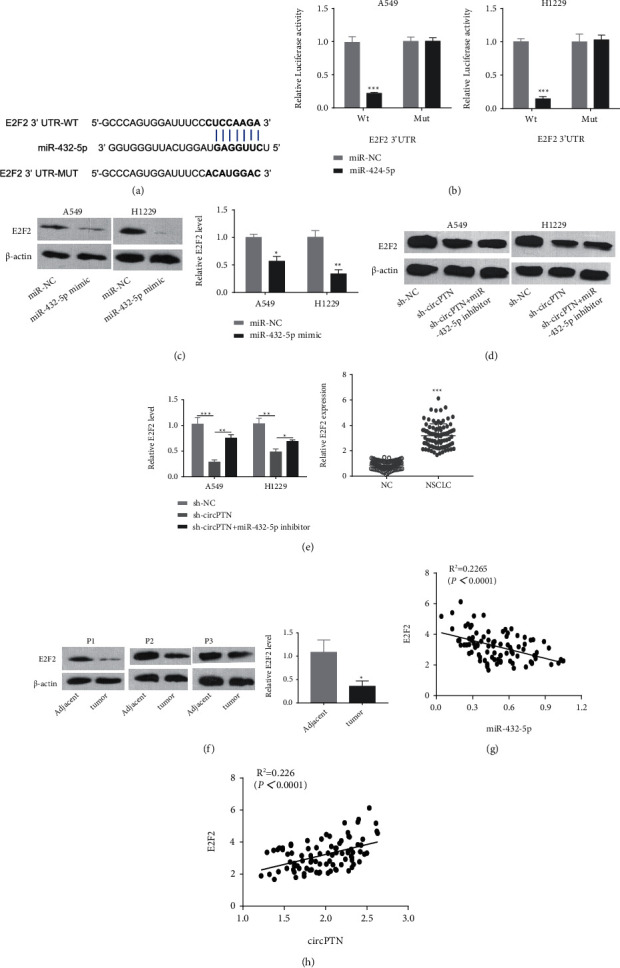
circPTN regulates E2F2 protein expression through sponge miR-432-5p. (a) The binding sites between miR-432-5p and E2F2 mRNA were predicted by TargetScan. (b) Dual-luciferase reporter assay in A549 and H1229 cells using WT reporter (with wildtype binding sites) or MUT reporter (with mutated binding sites) in the presence of miR-NC or miR-432-5p mimic. (c) The protein level of E2F2 in A549 and H1229 cells upon miR-432-5p mimic transfection was detected by WB. (d) E2F2 protein levels in A549 and H1229 cells transfected with sh-NC, sh-circTPN, or sh-circTPN and miR-432-5p inhibitor were determined by WB. (e) qRT-PCR was used to detect the expression level of E2F2 in 90 pairs of NSCLC tissues and adjacent normal tissues. (f) The protein levels of E2F2 protein in three pairs of NSCLC tissues and corresponding adjacent tissues were determined by WB. (g) The correlation between the expression levels of E2F2 and miR-432-5p in NSCLC tissues. (h) The correlation between the expression levels of E2F2 and circPTN in NSCLC tissues. ∗*P* < 0.05; ∗∗*P* < 0.01; and ∗∗∗*P* < 0.001.

To explore the downstream target of miR-432-5p, TargetScan software was employed to predict that there was binding sites in the 3' UTR of E2F2 mRNA for miR-432-5p (Figure 4(a)). miR-432-5p mimic significantly suppressed the luciferase activity of WT reporter compared with miR-NC, while inhibition was not abrogated when the predicted E2F2 binding sites were mutated (Figure 4(b)). miR-432-5p overexpression significantly reduced E2F2 protein level (Figure 4(c)). In addition, circPTN knockdown also reduced the E2F2 protein level and the co-transfection of miR-432-5p inhibitor partially restored E2F2 expression (Figure 4(d)). As revealed by qRT-PCR and WB assays, E2F2 showed a relatively higher expression in NSCLC tissues compared to the adjacent normal tissues (Figure 4(e) and 4(f)). Moreover, E2F2 expression was negatively correlated with miR-432-5p level, but positively correlated with circPTN in NSCLC tissues (Figure 4(g) and 4(h)). These data imply that circPTN regulates E2F2 expression through sponging miR-432-5p in NSCLC cells.

### 3.5. circPTN Regulates the Malignant Phenotype of NSCLC Cells through the miR-432-5p/E2F2 Axis

**Figure 5:**
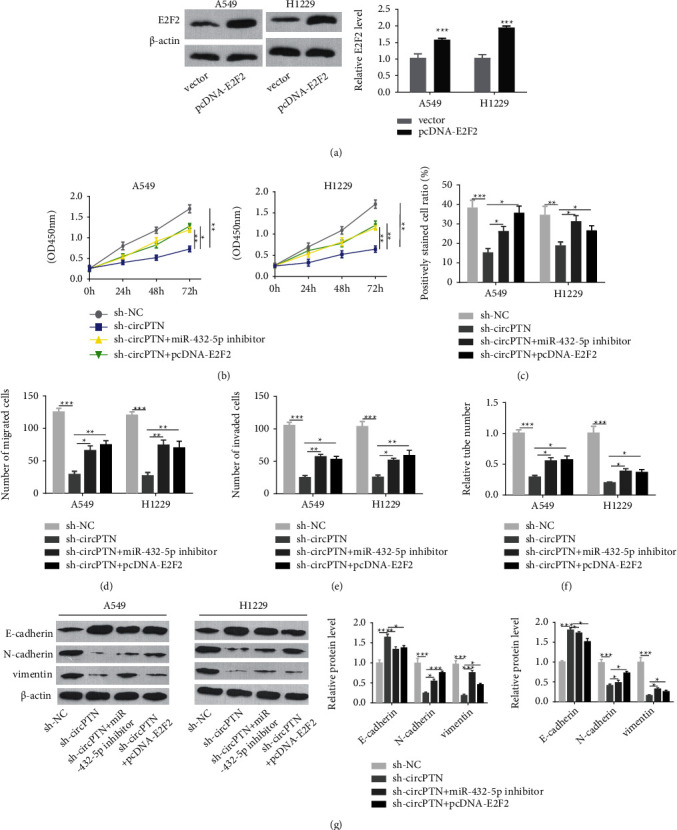
circPTN regulates the malignant phenotype of NSCLC cells through the miR-432-5p/E2F2 axis. (a) The protein levels of E2F2 in A549 and H1229 cells upon the transfection of pcDNA-E2F2 expression plasmid. (b) CCK-8 proliferation assay in A549 and H1229 cells with indicated treatment. (c) EdU incorporation assay in A549 and H1229 cells with indicated treatment. (d) Transwell-migration assay in A549 and H1229 cells with indicated treatment. (e) The transwell-invasion experiment in A549 and H1229 cells with indicated treatment. (f) The tube formation assay in A549 and H1229 cells with indicated treatment. (g) WB was performed to detect EMT-related proteins (E-cadherin, N-cadherin, and vimentin) in A549 and H1229 cells with indicated treatment. ∗*P* < 0.05; ∗∗*P* < 0.01; and ∗∗∗*P* < 0.001.

We next sought to validate the role of miR-432-5p/E2F2 axis in the effect of circPTN. We employed pcDNA-E2F2 expression vector, which could effectively promote the protein level of E2F2 in NSCLC cells (Figure 5(a)). CCK-8 proliferation assay and EdU staining assays showed that circPTN knockdown inhibited cell proliferation, which was partially rescued after the co-transfection of pcDNA-E2F2 plasmid or miR-432-5p inhibitor (Figure 5(b) and 5(c)). Similar rescue effects were observed using Transwell migration and invasion assays and tube formation assay in NSCLC cells (Figures 5(d)–5(f)). As revealed by WB assay, circPTN silencing suppressed the expression of mesenchymal markers (vimentin and N-cadherin) but increased the expression of E-cadherin. However, the co-transfection of pcDNA-E2F2 plasmid or miR-432-5p inhibitor partially reversed the effect of circPTN silencing (Figure 5(g)). Together, these data suggest that miR-432-5p/E2F2 axis mediates the role of circPTN in regulating the malignant phenotype of NSCLC cells.

### 3.6. Knockdown circPTN Inhibits Proliferation and Metastasis in NSCLC Cells In Vivo

**Figure 6:**
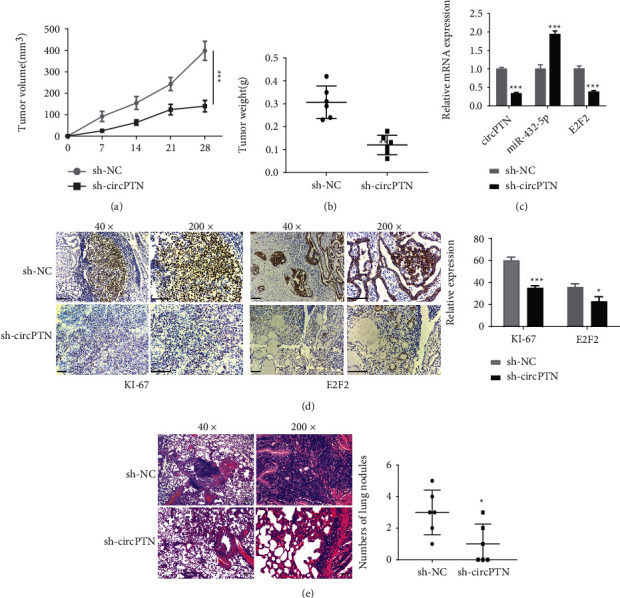
Knockdown of circPTN inhibits tumorigenesis and metastasis of NSCLC cells in vivo. (a) H1299 cells with sh-NC or sh-circTPN were inoculated subcutaneously in nude mice. Subcutaneous xenograft tumor size was measured every 7 days. (b) Subcutaneous tumor mass of H1299 cells in different groups was measured on day 28. (c) The expression levels of circPTN, miR-432-5p, and E2F2 in subcutaneous tumors of different groups were quantified by qRT-PCR. (d) The protein levels of Ki-67 and E2F2 in subcutaneous tumor tissues in different groups were detected by immunohistochemistry (IHC). (e) H&E staining was performed to examine pulmonary metastatic nodules in the lung tissues of the two groups. ∗*P* < 0.05; ∗∗*P* < 0.01; and ∗∗∗*P* < 0.001.

To evaluate the role of circPTN in tumorigenesis, we established subcutaneous xenograft growth model in nude mice using H1299 cells stably expressing sh-NC or sh-circPTN. circPTN knockdown significantly inhibited the tumor volume increase when compared with the sh-NC group (Figure 6(a)). In addition, knockdown of circPTN significantly lowered the tumor weight (Figure 6(b)). The retarded tumor growth in the sh-circPTN group was associated with a reduced E2F2 level and an increased miR-432-5p level in the xenograft tumors (Figure 6(c)). Based on IHC analysis, both Ki-67 (proliferation marker) and E2F2 levels were significantly suppressed in sh-circPTN group (Figure 6(d)). Moreover, relative to the sh-NC group, there were less pulmonary metastases in the lung tissues after circPTN knockdown (Figure 6(e)). Therefore, circPTN expression is required to support the tumorigenesis of NSCLC cells *in vivo*.

## 4. Discussion

CircRNAs have been proposed as cancer diagnostic biomarkers as they show superior stability over linear RNAs. Cancer-related circRNAs can be detected in plasma exosomes from nude mice bearing tumor xenografts [27]. In addition, EML4-ALK (one of the fusion genes in cancer) can also be detected in the plasma samples from NSCLC patients [28]. In this study, we reported a high-level expression of circPTN in NSCLC cells and tissues, which is associated with a poor prognosis in NSCLC patients. Furthermore, silencing circPTN impaired the malignant phenotype of NSCLC cells. These results highlight the potential of circPTN as a prognostic marker for NSCLC patients.

circRNAs could sponge miRNAs to regulate gene expression during tumor progression [7]. Through bioinformatics prediction and RNA pull-down experiment, miR-432-5p was identified as a downstream target of circPTN. Since circPTN and miR-432-5p also showed negative correlation in NSCLC cells, these data suggest circPTN sponges miR-432-5p and negatively regulates its activity in NSCLC cells. However, it remains to be investigated what the upstream mechanisms are underlying the upregulation of circPTN in NSCLC cells.

Previous studies have demonstrated that miR-432-5p acts as a tumor suppressor molecule that is frequently downregulated in various tumors, including pituitary adenomas [29], hepatocellular carcinoma [30], osteosarcoma [31], lung adenocarcinoma [32], and nasopharyngeal carcinoma [33]. Since circPTN sponges miR-432-5p and downregulates miR-432-5p, our data also suggest that miR-432 acts as a tumor-suppressor factor in NSCLC cells.

We further revealed that E2F2 is a downstream target of miR-432-5p. E2F2 is negatively correlated with miR-432-50 expression but positively correlated with circPTN expression. The overexpression of E2F2 also rescued the phenotype of NSCLC cells upon circPTN silencing. These data suggest that E2F2 acts as an oncogene, which is upregulated by circPTN and negatively regulated by miR-432-5p in NSCLC cells. These data seem consistent with the previously reported oncogenic role of E2F2 in different cancers [34–36]. E2F2 belongs to the E2F transcription factor family that binds to DNA with DPDP1-polypeptide at the E2 recognition site in gene promoter region, thus producing the gene expressions related to DNA replication and cell cycle progression [35]. E2F2 plays a critical role in promoting the progression of the cell cycle [37]. In this context, E2F2 overexpression can be an important factor contributing to the accelerated cell cycle progression, which predicts dismal overall survival in NSCLC patients [31]. Indeed, its overexpression could rescue the retarded proliferation in NSCLC cells upon circPTN silencing. Overall, these data support the notion that E2F2 acts as an oncogenic transcription factor downstream of circPTN, which promotes proliferation and tumor progression in NSCLC cells.

## 5. Conclusion

In summary, our results revealed the upregulation of circPTN in NSCLC tumors and its contribution to the malignant phenotype of NSCLC cells. circPTN maintains the expression of E2F2 in NSCLC cells by sponging miR-432-5p. Since a high level of circPTN is correlated with the poor prognosis in NSCLC patients, circPTN may serve as a prognostic marker in NSCLC.

## Figures and Tables

**Table 1:** Correlations of circPTN expression level with clinicopathologic features of NSCLC patients.

Factor		circPTN expression		*P*-value
		Low (*n* = 45)	High (*n* = 45)	
Gender				0.5204
	Male	28	25	
	Female	17	20	
Age				0.1365
	>55	23	16	
	≤55	22	29	
Tumor size				0.3522
	>4 cm	11	15	
	≤4 cm	34	30	
TNM				0.0203
	I/II	29	18	
	III/IV	16	27	
Lymph node metastasis				0.0328
	Absence	24	14	
	Presence	21	31	

## Data Availability

The datasets used and/or analyzed during the current study are available from the corresponding author via email request.
